# The Efficacy and Safety of Early Renal Replacement Therapy in Critically Ill Patients With Acute Kidney Injury: A Meta-Analysis With Trial Sequential Analysis of Randomized Controlled Trials

**DOI:** 10.3389/fmed.2022.820624

**Published:** 2022-02-21

**Authors:** Chuan Xiao, Jingjing Xiao, Yumei Cheng, Qing Li, Wei Li, Tianhui He, Shuwen Li, Daixiu Gao, Feng Shen

**Affiliations:** ^1^Department of Intensive Care Unit, The Affiliated Hospital of Guizhou Medical University, Guiyang, China; ^2^School of Clinical Medicine, Guizhou Medical University, Guiyang, China

**Keywords:** mortality, early renal replacement therapy, delayed renal replacement therapy, acute kidney injury, trial sequential analysis (TSA)

## Abstract

The efficacy and safety of early renal replacement therapy (eRRT) for critically ill patients with acute kidney injury (AKI) remain controversial. Therefore, the purpose of our study was to perform an up-to-date meta-analysis with the trial sequential analysis (TSA) of randomized controlled trials (RCTs) to evaluate the therapeutic effect of eRRT on patients in an intensive care unit (ICU). We extensively searched MEDLINE, EMBASE, LILACS, the Cochrane Central Register of Controlled Trials and ClinicalTrials.gov, Gray Literature Report, and Bielefeld Academic Search Engine (BASE), and conducted an updated search on December 27, 2021. The included studies were RCTs, which compared the efficacy and safety of eRRT and delayed renal replacement therapy (dRRT) on critically ill patients with AKI. We adopted TSA and sensitivity analysis to strengthen the robustness of the results. About 12 RCTs with a total of 5,423 participants were included. Patients receiving eRRT and dRRT had the similar rate of all-cause mortality at day 28 (38.7% vs. 38.9%) [risk ratio (RR), 1.00; 95%CI, 0.93–1.07, *p* = 0.93, *I*^2^ = 0%, *p* = 0.93]. A sensitivity and subgroup analysis produced similar results for the primary outcome. TSA showed that the required information size was 5,034, and the cumulative *Z*-curve crossed trial sequential monitoring boundaries for futility. Patients receiving eRRT had a higher rate of renal replacement therapy (RRT) (RR, 1.50, 95% CI: 1.28–1.76, *p* < 0.00001, *I*^2^ = 96%), and experienced more adverse events comparing to those receiving dRRT (RR: 1.41, 95% CI: 1.22–1.63, *p* < 0.0001, heterogeneity not applied). The most remarkable and important experimental finding is that, to our knowledge, the current meta-analysis included the largest sample size from the RCTs, which were published in the past 10 years to date, show that eRRT had no significant survival benefit for ill patients with AKI compared with dRRT and TSA indicating that no more studies were needed to confirm it.

**Trial Registration:**

INPLASY, INPLASY2020120030. Registered 04 December 2020.

## Introduction

Acute kidney injury (AKI) is an important complication in patients who are admitted to an intensive care unit (ICU) and is associated with a high risk of death (1). AKI could result from prerenal azotemia, acute tubular necrosis, or post-renal obstructive disease (2). Renal replacement therapy (RRT) is an effective treatment for AKI when indicated (3), helping to optimize fluid administration and hemodynamic management, etc. (4–6). Therefore, RRT is often applied whenever patients suffered from fluid overload and/or other complications [7–9] (7, 8).

However, there is no optimal timing of RRT, and whether early RRT (eRRT) is superior to delayed RRT (dRRT) is also a matter of controversy (9–11). Some multicenter, randomized controlled trials (RCTs) indicated that eRRT has not shown clinical benefits (12, 13), but others demonstrated that eRRT significantly reduces the mortality of patients with AKI (6, 14).

In view of controversial results, recently, two high-quality RCTs were published in NEJM (15) and Lancet (16), which compared the effects of eRRT vs. dRRT on critically ill patients with AKI. So, we did an updated systematic review involving sufficient patients from RCTs to explore the uncertainty arising from conflicting results. Review evidence may provide contemporary evidence to guide clinical policies and practices.

In the traditional meta-analysis, the positive results are easy to be changed after more evidence is accumulated (17). The trial sequential analysis (TSA), however, has the potential to make conclusions more reliable than traditional meta-analyses (18, 19). TSA can also provide “futility boundaries” to identify invalid conclusions earlier, preventing researchers from spending a lot of resources on unneeded research. Here, we performed TSA to minimize random errors in a cumulative meta-analysis and strengthen the robustness of the results.

## Methods

### Overview

Our systematic review was registered at INPLASY (INPLASY2020120030), (https://inplasy.com/inplasy-2020-12-0030/), following a prespecified protocol and complying with the Preferred Reporting Items for Systematic Meta-Analyses (PRISMA) guideline (20).

### Eligibility Criteria

We included studies, which met all the following criteria: (1) RCTs published from January 1, 2010, to October 11, 2020, and an updated search was conducted on December 27, 2021. (2) Subjects were critically ill patients (≥18 years) with AKI (KDIGO stage 2 or 3, or at the failure stage of RIFLE) (21). (3) With a clear criterion used for defining eRRT and dRRT.

### Search Strategy

We did an extensive electronic search from January 1, 2010, to October 11, 2020, based on the recommendations (20, 22) from MEDLINE, EMBASE, LILACS, the Cochrane Central Register of Controlled Trials, and ClinicalTrials.gov, and conducted an updated search on December 27, 2021. Besides, we searched the two gray literature databases: The Grey Literature Report (www.greylit.org/) and Bielefeld Academic Search Engine (BASE) (http://www.base-search.net/about/en/). The detailed retrieval method was included as Supplementary Materials.

### Selection of Studies

The two authors independently screened research titles and abstracts that were potentially relevant to this article according to the inclusion criteria. To determine studies that meet the inclusion criteria of this study, we retain potentially relevant data and research information and evaluate the full text when necessary. Any disagreements in the selection of studies were negotiated or consulted with a third review author if necessary.

### Data Extraction and Effect Measure

Data were independently entered into an Excel spreadsheet by three reviewers. We collected the data including first author, recruitment period, the year of publication, sample size, mean age, gender, RRT modality, primary outcome: all-cause mortality at day 28, secondary outcomes: all-cause mortality at days 60 and 90, all-cause mortality in an ICU and a hospital, the length of stay (LOS)in an ICU and a hospital, ventilator-free days vasoactive agents free days/rate of RRT dependence at day 28, and the risk of total adverse events and specific adverse events (bleeding, arrhythmia, and hypotension). Any disagreements in data extraction were negotiated or consulted with a third review author if necessary. For binary classification results, the risk ratio (RR) with 95% CI was used to describe the binary classification results, and the mean difference (MD) with 95% CI was used to describe continuous variables.

### Risk of Bias Assessment and Certainly Assessment

At least two review authors independently evaluated the methodological quality of each included trial and assessed the risk of bias according to the Cochrane Handbook for Systematic Reviews of Interventions (22). Included trials were judged for the risk of bias in all bias domains (23). Any disagreements concerning the assessment of the risk of bias were negotiated with a third review author if necessary. We assessed the certainty of the evidence for all-cause mortality at day 28, the LOS of survivors in an ICU and a hospital, the rate of RRT dependence at day 28, and the incidence of total adverse events by grading with Grading of Recommendations, Assessment, Development, and Evaluations (GRADE) pro GDT (24) available from gdt.gradepro.org.

### Reporting Biases Assessment

Visual inspection of funnel plots and contour-enhanced funnel in the graphic was used to assess the potential existence of reporting bias. Besides, Begg's and Egger's tests were performed to quantitatively assess the publication bias.

### Data Analysis

We undertook this systematic review following the recommendations of the Cochrane Handbook for Systematic Reviews of Interventions (22) and Meta package for the R language. We performed a meta-analysis for the outcomes with comparable methods in a similar population when more than one trial was included by using statistical software Review Manager 5. We set *p* < 0.05 as the statistical significance and represented individual trial and summary reports with 95% CIs. Moreover, we evaluated the statistical heterogeneity of each outcome through the *I*^2^ statistic measure and chi-squared test, in which *p* ≤ 0.1 was regarded as to be significant. We adopted a random-effects model in all analyses because of fears of that clinical heterogeneity and methodological heterogeneity. To explore the eventual sources of heterogeneity, we conducted the respective subgroup analyses based on these characteristics including study design, patient population, the modality of RRT, and the presence of sepsis at randomization for the primary outcome. We also performed sensitivity analyses for the primary outcome such that investigating the potential effect of excluding studies judged as being at unclear or a high risk of bias in any of the risk of bias domains. Furthermore, we implemented the two important analyses, the “best-worst-case” scenario and the “worst-best-case” scenario (25) to assess the possible impact of missing data on the primary outcome.

### Trial Sequential Analyses

Recent studies showed that TSA has the potential to make conclusions more reliable than those traditional meta-analyses (18, 26). Also in traditional meta-analyses, it is sometimes to get positive conclusions resulting from “random error” rather than a “real” intervention effect (27). In this situation, the TSA software provides methods to minimize the incidences of these false positive results (28). TSA is also able to provide “futility boundaries” that could draw invalid conclusions early (27, 29). In our analysis, we conducted TSA using the TSA software (version 0.9.5.10 Beta) (19), of which the information size was calculated according to an anticipated—relative risk reduction (RRR) of 15% with a power of 90% (15, 30) and a type-I error value of 1% (two-sided).

## Results

### Research Results

**Figure 1:**
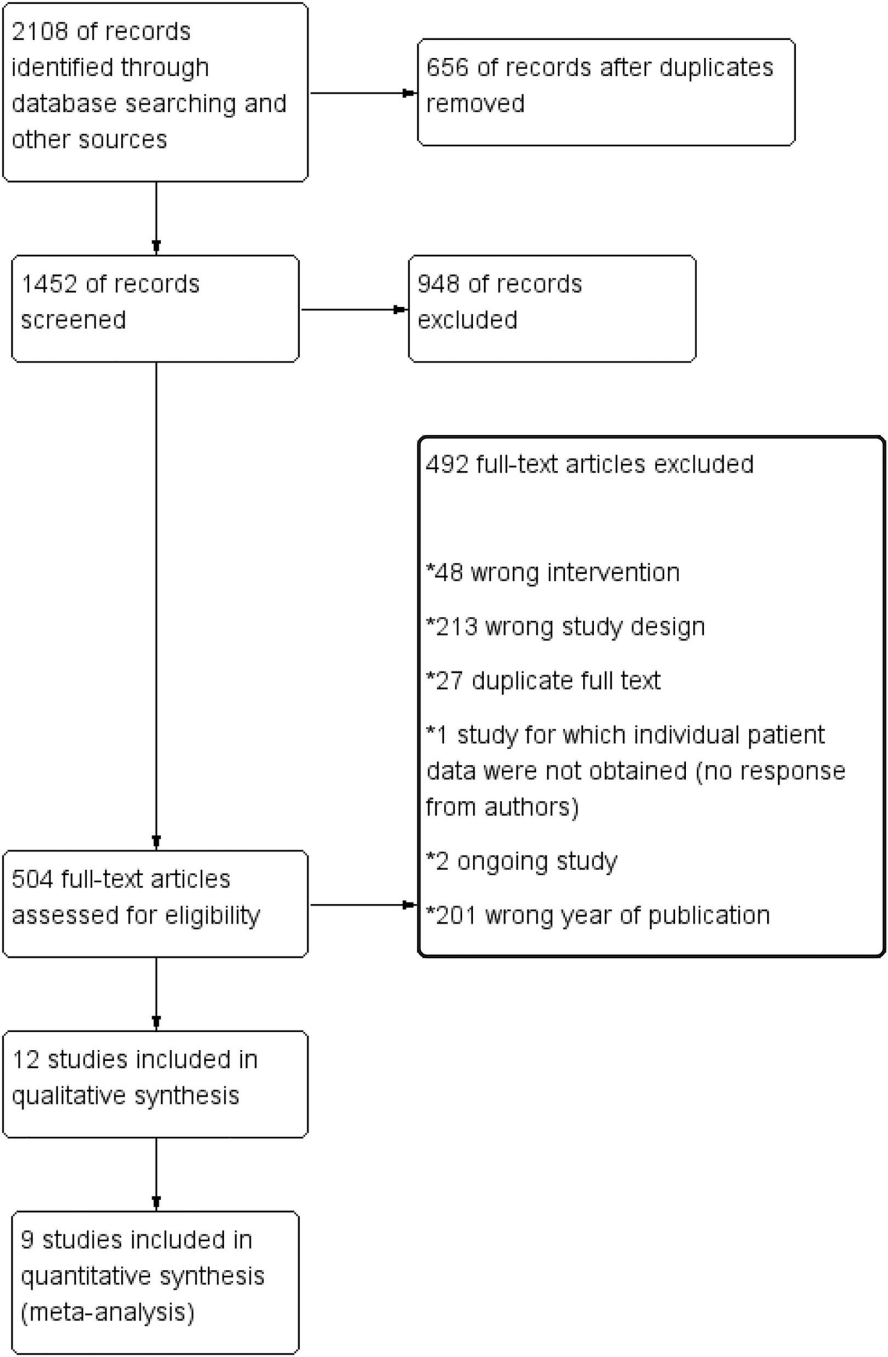
The flow diagram of retrieved and included records.

We totally screened 2,108 titles and abstracts of which 504 full-text reports were assessed for eligibility and 492 references were excluded due to some reasons (Figure 1). About 12 records were finally included for a meta-analysis and 9 of them were selected in quantitative synthesis for the primary outcome in our meta-analysis (Figure 1).

### Characteristics of the Studies

About 12 RCTs fulfilled the planned inclusion criteria. The baseline characteristics of the included studies are presented in Table 1. Around 9 of 12 studies came from a multi-center (12, 13, 15, 16, 30–34), while the other 3 came from a single-center (6, 35, 36). Severity scores of diseases reported mostly were Sequential Organ Failure Assessment (SOFA) and Acute Physiology and Chronic Health Evaluation (APACHE II).

**Table 1:** Baseline characteristics of the randomized controlled clinical trials included in the meta-analysis.

**Author**	**Year**	**Country**	**Design setting and Population**	**Median time of RRT initiation (IQR), h**		**No. of patients**		**Female (%)**		**Mean population age, years (SD)**		**SOFA(SD)**		**APACHEII (SD)**		**RRT** **modality**	**Missing** **primary outcome**
				**eRRT**	**dRRT**	**eRRT**	**dRRT**	**eRRT**	**eRRT**	**eRRT**	**dRRT**	**eRRT**	**dRRT**	**eRRT**	**dRRT**		
Sean M	2020	Nationwide	Multicenter mixed population	6.1 (3.9,8.8)	31.1 (19.0,71.8)	1465	1462	470/1465 (31)	467/1462 (32)	65(14)	65(13)	12(4)	12(4)	NA	NA	CRRT	No
Gaudry	2021	France	Multicenter mixed population	33 (24,60)	3 (2,5)	137	141	35 (26)	38 (27)	65 (13)	65(12)	NA	NA	NA	NA	IHD, CRRT Mixed	No
Gaudry	2016	France	Multicenter mixed population	2 (1, 3)	57 (25,83)	311	308	102/311 (32.7)	110/308 (35.7)	65(14)	67(13)	NA	NA	NA	NA	IHD, CRRT	No
Barbar	2018	France	Multicenter mixed population	7.6 (4.4,11.5)	51.5 (34.6,59.5)	246	242	104/246 (42.2)	88/242 (36.3)	69(12)	69(13)	NA	NA	NA	NA	CRRT IHD Mixed	No
Lumlertgul	2018	Thailand	Multicenter mixed population	2 (1, 3)	21 (17,49)	58	60	29/58 (50)	31/60 (52)	68(15)	67(17)	13(3)	11(4)	24.5(6.4)	21.8(6.9)	CRRT	No
Zarbock	2016	Germany	Single-center Surgical population	6 (4, 7)	25.5(18.8)40.3)	112	119	34/112 (30.3)	51/119 (42.9)	66(14)	68(13)	16(2)	16(2)	30.6(7.5)	32.7(8.8)	CRRT IHD MIXED	No
XIA	2019	China	Single-center mixed population	NA	NA	30	30	15/30 (50)	12/30 (30)	65(12)	67(11)	10(3)	10(3)	19.25(3.43)	18.34(3.27)	CRRT	No
Srisawat	2017	Thailand	Multicenter. mixed population	NA	NA	20	20	NA	NA	NA	NA	NA	NA	NA	NA	CRRT	No
Tukaram E	2013	India	Single-center medical population	NA	NA	102	106	40/102 (39.2)	27/106 (25.5)	43(15)	42(16)	8(3)	8(3)	NA	NA	IHD	Yes
Vaara	2014	Finnish	Multicenter mixed population	3.3(2.0–7.4)	35.5(22.7–59.8)	90	44	31/90 (34.4)	23/44 (52.3)	64(11)	67(16)	12(4)	12(4)	NA	NA	CRRT	Yes
Wald	2015	Canada	Multicenter mixed population	7.4 (6.1,9.6)	31.6 (22.8,59.5)	48	52	13/48 (27.1)	15/52 (28.8)	62(12)	64(14)	13(3)	13(3)	NA	NA	IHD, SLED, CRRT	Yes
jun	2014	Australia New Zealand.	Multicenter mixed population	NA	NA	109	111	36/109 (33)	36/111 (32)	66(13)	64(15)	2(1)	2(2)	NA	NA	CRRT	No

*APACHE II, Acute Physiology and Chronic Health Evaluation II; SOFA, Sequential Organ Failure Assessment; CRRT, continuous renal replacement therapy; IHD, Intermittent hemodialysis; SLED, Sustained low-efficiency dialysis; HCO-CVVHD, high cut-off continuous veno-venous hemodialysis; NA, not available*.

The criteria for eRRT and dRRT of the included trials are depicted in Table 2. In the RENAL study by Jun et al. (31), as 439 patients were assigned to four groups according to variant initiating time of CRRT other than to early and delay initiation, we only selected the patients from the earliest (within 7.1 h of AKI diagnosis) and latest group (46 h after AKI diagnosis). Therefore, only 220 of 439 patients were included for analysis. In a trial by Lumlertgul et al. (13), the furosemide stress test (FST) was performed for all patients with AKI, FST-nonresponsive patients were then randomized to eRRT or dRRT, and 118 of 162 patients were included for analysis. In the trials by Srisawat et al. (34) and Xia et al. (36), patients were grouped based on the level of plasma neutrophil gelatinase-associated lipocalin (pNGAL), and only patients with a high level of pNGAL were assigned to receive either eRRT or dRRT, resulting in the inclusion of 40 of 60 patients from Srisawat's trial and of 60 of 100 patients from Xia's trial in our analysis.

**Table 2:** Definitions of early and delayed renal replacement therapy (RRT) in individual studies.

**Author**	**Year**	**Early initiation Criteria**	**Delayed initiation Criteria**
Gaudry (AKIK2)	2021	Within 12 h after fulfilling the randomization criteria.	Severe hyperkalemia (>6 mmol/l); hyperkalemia (>5.5 mmol/l) persisting despite medical treatment; Severe metabolic acidosis (pH <7·15); Severe pulmonary oedema; serum urea >40 mmol/L for 1 day.
Sean (STARRT-AKI)	2020	Within 12 h after fulfilling the randomization criteria.	Severe hyperkalemia (>6 mmol/l); Severe metabolic acidosis (pH <7·2); severe pulmonary oedema; persistent AKI for at least 72 h after randomization.
Gaudry (AKIK)	2016	Within 6 h after documentation of AKI stage 3 of KDIGO classification	Severe hyperkalemia (>6 mmol/L); severe pulmonary, oedema refractory to diuretics; severe acidosis (pH <7·15); serum urea >40 mmol/L; oligo-anuria >72 h
Barbar (IDEAL-ICU)	2018	Within 12 h after the onset of acute kidney injury that was determined to be at the failure stage of the risk, injury, failure, loss, and end-stage kidney disease (RIFLE) classification	Severe hyperkalemia (>6·5 mmol/L); severe pulmonary oedema refractory to diuretics; severe metabolic acidosis (pH <7·15); no renal function recovery after 48h
Lumlertgul (FST)	2018	Within 6 h of randomization	Serum urea ≥100 mg/dL; Severe hyperkalemia (>6 mmol/L); Severe metabolic acidosis (pH <7·15); Severe pulmonary oedema
Zarbock (ELAIN)	2016	Within 8 h of stage 2 AKI diagnosed	Within 12 h of stage 3 AKI indicated
XIA	2019	Performed as soon as possible	Severe hyperkalemia (>6·5 mmol/L); Severe pulmonary oedema; Severe metabolic acidosis (pH <7·20)
Srisawat	2017	A session of CRRT was run within 12 h after group assignment for early RRT group	Severe acidosis (Ph <7.2), severe peripheral edema, pulmonary edema, no response to diuretics, refractory hyperkalemia (>6·5 mmol/L or the presence of ECG change: tall T wave, absent P wave, or wide QRS wave), anuria or oliguria, or high BUN level>60 mg/dL.
Tukaram E	2013	Serum urea nitrogen level increased to 70 mg/dL and/or creatinine level increased to 7 mg/dL irrespective of complications	Treatment-refractory hyperkalemia, volume overload, and acidosis. Uremic nausea and anorexia leading to inability to maintain nutrient intake also were indications to initiate dialysis therapy
Vaara	2014	Within 12 h from manifestation of indications	RRT initiated 12 h after indications: hypercalcemia, severe acidosis, plasma urea.36 mmol/L, oliguria, or anuria, fluid overload with pulmonary edema
Wald	2015	Within 12 h from eligibility	Severe hyperkalemia (>6 mmol/L); severe pulmonary oedema; severe metabolic acidosis (serum bicarbonate <10 mmol/L)
Jun (RENAL)	2014	Within 7.1h of AKI diagnosed according to RIFLE criteria	After 46h of AKI diagnosed according to RIFLE criteria

*KDIGO^22^, kidney disease: improving global outcomes; RIFLE^44^, risk, injury, failure, loss, and end-stage kidney disease*.

*KDIGO 1: 1.5–1.9 times baseline or UOP 26.5 umol/L (0.3 mg/dl) increase in creatinine within 48 or UOP <0.5 ml/kg/h for 6–12 h*.

*KDIGO 2:2.0–2.9 times baseline increases in creatinine or UOP <0.5 ml/kg/h for > 12 h*.

*KDIGO 3: 3.0 times baseline or creatinine ≥ 354 umol/L (4.0 mg/dl) or UPO <0.3 ml/kg/h for > 24 h or anuria for ≥ 12 h*.

*AKI, Acute renal injury*.

About 12 RCTs with a total of 5,423 patients were included in a meta-analysis, of whom 2,728 received eRRT and other 2,695 received dRRT. The basic characteristics of 5,423 patients were detailed in Table 3. These patients had similar age, sex ratio, serum creatinine, blood urea nitrogen, and SOFA score in eRRT and dRRT groups.

**Table 3:** Characteristics of patients from the randomized controlled trials (RCTs) included in the meta-analysis.

	**eRRT group**	**dRRT group**	95% CI^*^	***P***
Age	64.3 ± 14.6	64.7 ± 14.4	−0.04(−0.10,0.02)	0.23
Female (%)	918(33.6%)	898 (33.5%)	1.00(0.90,1.12)	0.97
SOFA score	11.2 ± 4.1	11.3 ± 4.1	−0.01(−0.28,0.29)	0.24
Serum creatinine(mg/dl)	3.7 ± 2.3	3.6 ± 2.2	−0.24(−0.49,0.08)	0.25
Blood urea nitrogen (mg/dL)	58.6 ± 32.2	62.1 ± 32.0	−5.45(−14.39,3.49)	0.23

*Data are presented as means ± SD or n/N (%)*.

*SOFA, Sequential Organ Failure Assessment*.

* *For binary classification results, the risk ratio (RR) with 95% CI was used to describe the binary classification results, and the mean difference (MD) with 95% CI was used to describe continuous variables*.

### Quality of Studies

**Figure 2:**
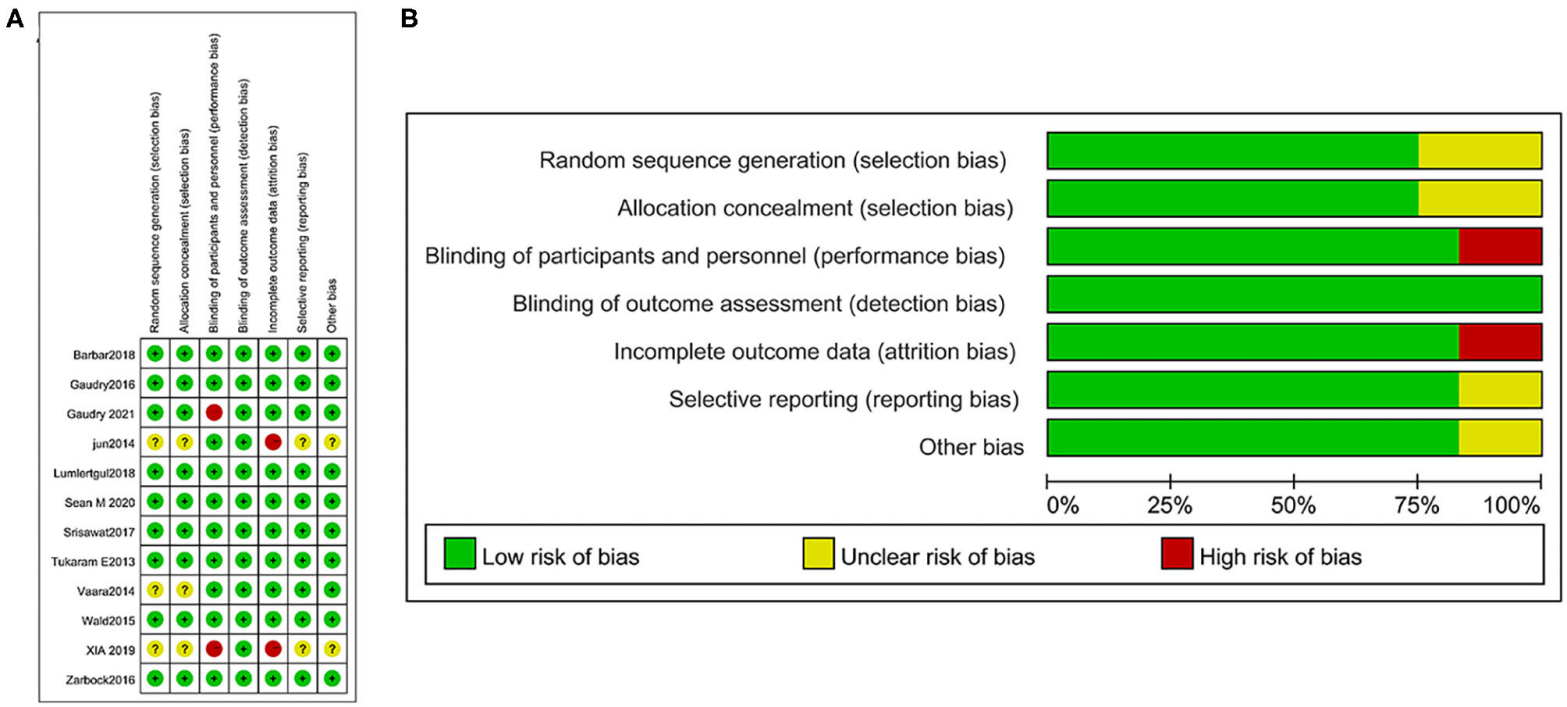
Risk of bias summary: a review of authors' judgments concerning the risk of bias in the included studies **(A)** and risk of bias graph: a review of authors' judgments about each risk of bias item presented as percentages across all included studies **(B)**.

We evaluated the risk of bias for each included article following the Cochrane ROB (risk of bias) tool (Figure 2A) and graphed the degrees of risk of bias (Figure 2B). Of the included studies, 8 (67%) studies were judged to have a low risk of bias, and 3 (25%) were judged to have a high risk of bias, while 1 study (8%) was unclear.

### Certainty of the Evidence

Six main outcomes were graded by the GRADE for the quality of evidence (Table 4). The results of the analysis showed that the certainty of the evidence was high for the mortality at day 28, the rate of RRT, moderate for the LOS of survivors in an ICU, the LOS survivors in a hospital, and total adverse events. Meanwhile, the evidence for RRT dependence at day 28 was of low certainty due to serious inconsistency and imprecision.

**Table 4:** Summary of findings.

**Early RRT compared to Delayed RRT for critically ill patients with acute kidney injury**						
**Patient or population: critically ill patients with acute kidney injury** **Setting: Critical care** **Intervention: Early RRT**						
**Outcomes**	Anticipated absolute effects^*^ **(95% CI)**		**Relative** **effect** **(95% CI)**	**No of** **participants** **(studies)**	**Certainty of the** **evidence** **(GRADE)**	**Comments**
	**Risk with** **Delayed** **RRT**	**Risk with Early** **RRT**				
28 -day mortality	389 per 1,000	389 per 1,000 (362 to 417)	RR 1.00 (0.93 to 1.07)	4981 (9 RCTs)	⊕⊕⊕⊕ HIGH	
the rate of RRT	633 per 1,000	950 per 1,000 (810–1,000)	RR 1.50 (1.28–1.76)	5069 (10 RCTs)	⊕⊕⊕⊕ HIGH	
The rate of RRT-dependence at day 28	154 per 1,000	123 per 1,000 (77–198)	RR 0.80 (0.50–1.29)	1229 (7 RCTs)	⊕⊕○○ LOW	
LOS of hospital in survivors	-	MD **2.74 lower** (4.93 lower−0.55 lower)	-	4365 (5 RCTs)	⊕⊕⊕○ MODERATE	
LOS of Icu in survivors	-	MD **1.55 lower** (3.08 lower−0.02 lower)	-	4365 (5 RCTs)	⊕⊕⊕○ MODERATE	
Total adverse events	199 per 1,000	280 per 1,000 (243–324)	RR 1.41 (1.22–1.63)	3027 (2 RCTs)	⊕⊕⊕○ MODERATE	

* *The risk in the intervention group (and its 95% CI) is based on the assumed risk in the comparison group and the relative effect of the intervention (and its 95% CI). CI: confidence interval; RR: risk ratio; MD: mean difference*.

*GRADE Working Group grades of evidence*.

*High certainty: We are very confident that the true effect lies close to that of the estimate of the effect*.

*Moderate certainty: We are moderately confident in the effect estimate: The true effect is likely to be close to the estimate of the effect, but there is a possibility that it is substantially different*.

*Low certainty: Our confidence in the effect estimate is limited: The true effect may be substantially different from the estimate of the effect*.

*Very low certainty: We have very little confidence in the effect estimate: The true effect is likely to be substantially different from the estimate of effect*.

### Quantitative Data Synthesis

#### Primary Outcomes

**Figure 3:**
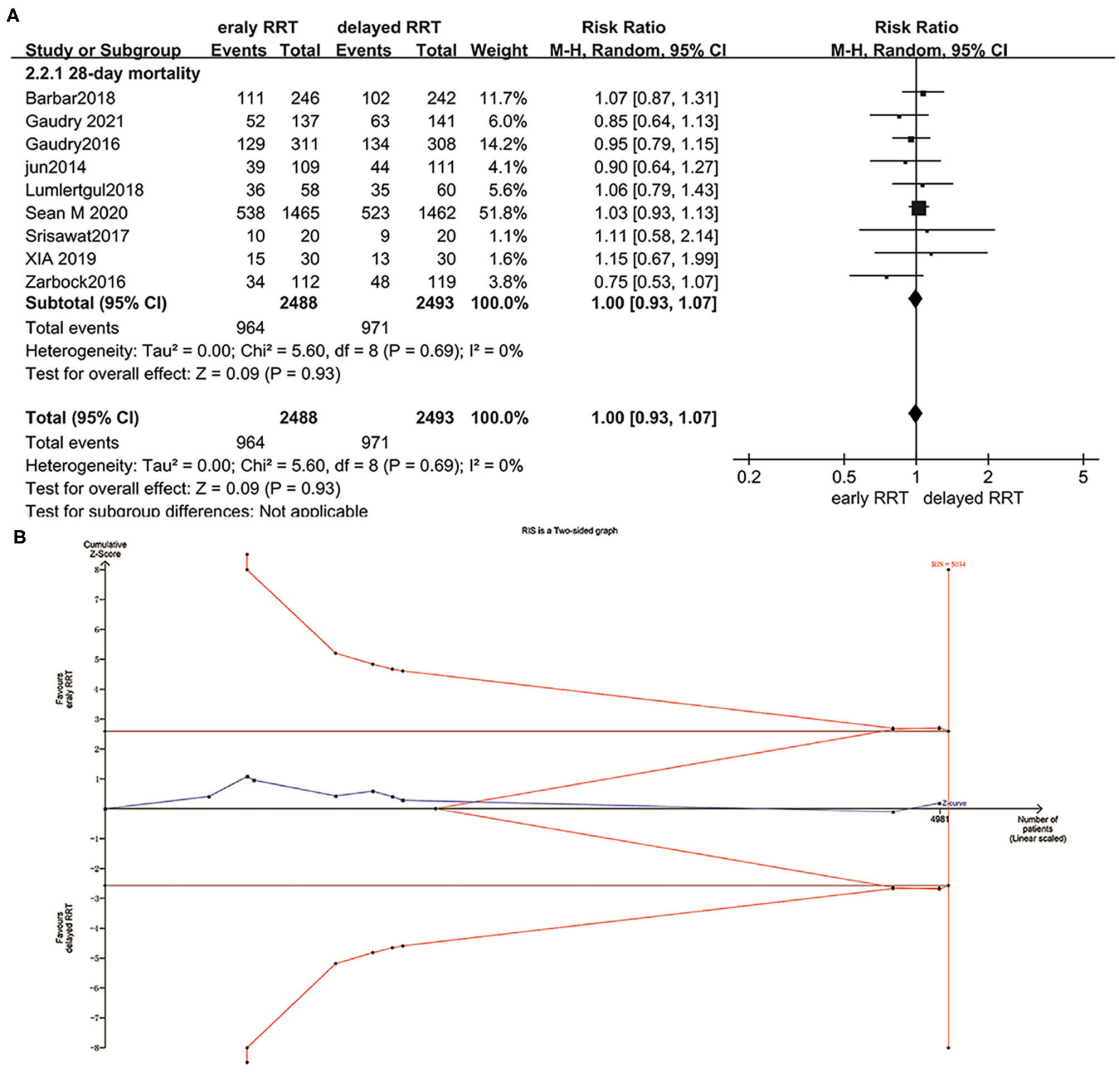
Forest plot for all-cause mortality at day 28 **(A)** and trial sequential analysis (TSA) for all-cause mortality at day 28 **(B)**.

About 9 of 12 trials with a total of 4,981 participants and a mean follow-up of 28 days reported all-cause mortality (6, 12, 13, 15, 16, 30, 34, 36). About 442 of 5,423 (8.1%) patients did not have complete data on the prespecified primary outcome. For the remaining 4,981 patients, the rates of all-cause mortality at day 28 (38.7% in eRRT and 38.9% in dRRT) were not significantly different between the two groups (RR 1.00, 95% CI: 0.93–1.07, *p* = 0.93) (Figure 3A). There was no inconsistency factor across trials, indicating a statistical heterogeneity (*I*^2^ = 0%, *p* = 0.69).

#### Reporting Biases for the Primary Outcome

**Figure 4:**
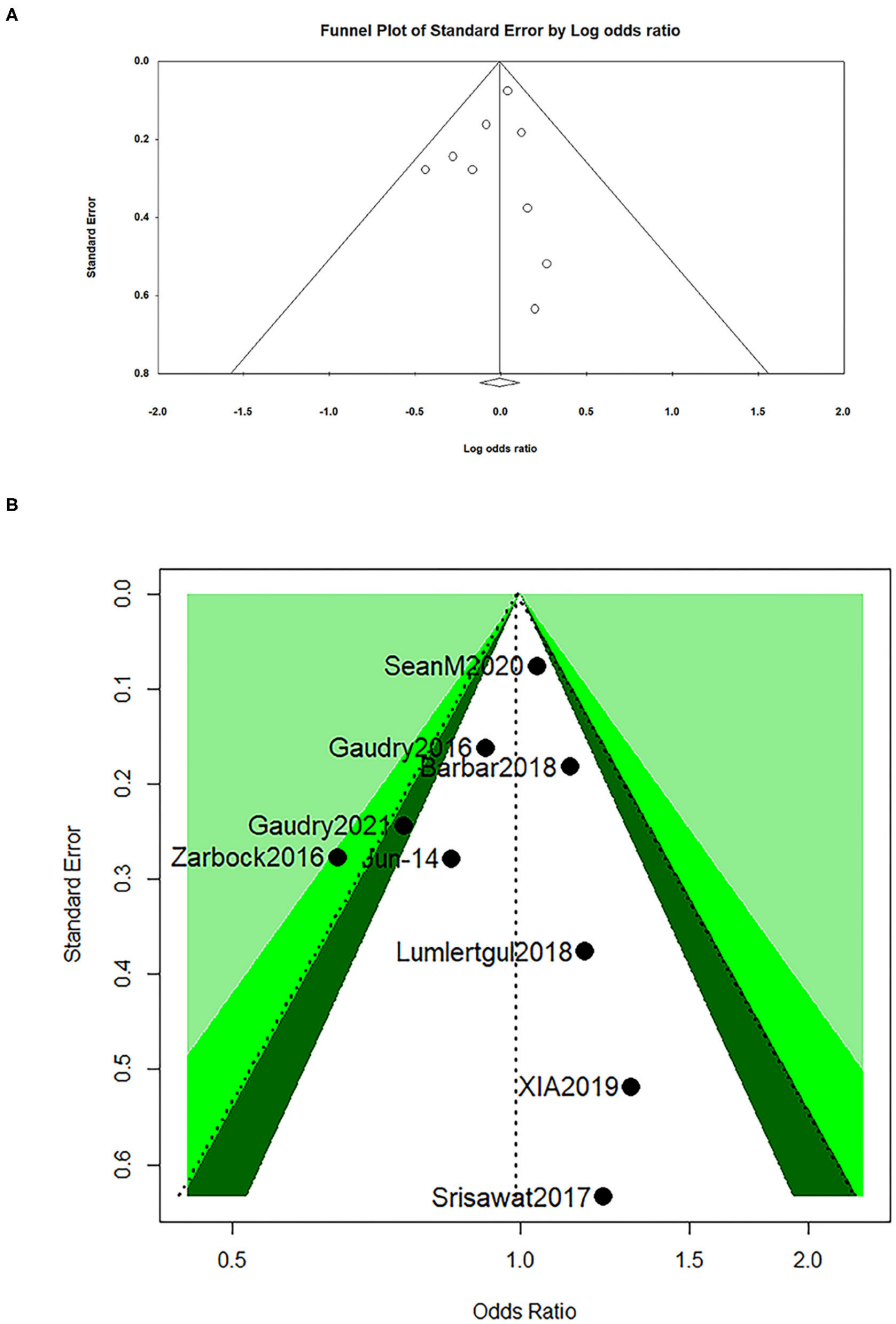
Funnel plot for all-cause mortality at day 28 **(A)** and contour-enhanced funnel for all-cause mortality at day 28 **(B)**.

The visual assessment showed that there was a partial asymmetry in the funnel plot and contour-enhanced funnel (Figures 4A,B), while *p*-values (0.51 and 0.75) were found in the Egger's test and Begg's test in a dominant model, respectively, which indicated that no statistical significance was found (Table 5).

**Table 5:** Begg's and Egger's tests to assess the publication bias.

**Tests**	***P***
Begg's 1-tailed	0.37
Begg's 2-tailed	0.75
egger's 1-tailed	0.25
egger's 1-tailed	0.51

#### A Subgroup Analysis for the Primary Outcome

To investigate heterogeneity, we performed a subgroup analysis for the primary outcome. As depicted in Table 6, there was no substantial difference between the groups regarding mortality at day 28 across prespecified subgroups based on study design, patient population, the modality of RRT, and the presence of sepsis at randomization for the primary outcome.

**Table 6:** Subgroup analysis for all-cause mortality at day 28.

**Subgroup**	**No of study**	**No of patients**		**Risk ratio (Random-effect model)**	**95% CI**	***I* ^2^**	***P***
		**eRRT**	**dRRT**				
**Patient population**							
≥100	8	2,458	2,463	0.99	0.93–1.07	0	0.88
<100	1	30	30	1.15	0.67–1.99	NA	0.61
**RRT modality**							
IHD	0						
CRRT only	4	217	221	1.02	0.84–1.24	0	0.83
Mixed	5	2,271	2,272	0.98	0.90–1.07	15	0.72
**Presence of sepsis at randomization**							
100%	1	246	242	1.07	0.87–1.31	NA	0.51
<100%	8	2,242	2,251	0.99	0.92–1.06	0	0.74
Study Design							
Single-center	2	142	149	0.89	0.59–1.33	40	0.56
Multi-center	7	2,346	2,344	1.01	0.94–1.08	0	0.88

*A subgroup analysis based on the following items: reason for admission, presence of sepsis at randomization, patient population, RRT modality, sample size, study design*.

*eRRT, early renal replacement therapy; dRRT, delayed renal replacement therapy; CRRT, continuous renal replacement therapy; IHD, intermittent hemodialysis*.

#### TSA for the Primary Outcome

The trial sequential analysis showed that the required information size was 5,034 and the cumulative *Z*-curve crossed trial sequential monitoring boundaries for futility (Figure 3B), indicating that there is no difference between the eRRT and dRRT in terms of mortality at day 28 and no more studies were needed.

#### A Sensitivity Analysis for the Primary Outcome

**Figure 5:**
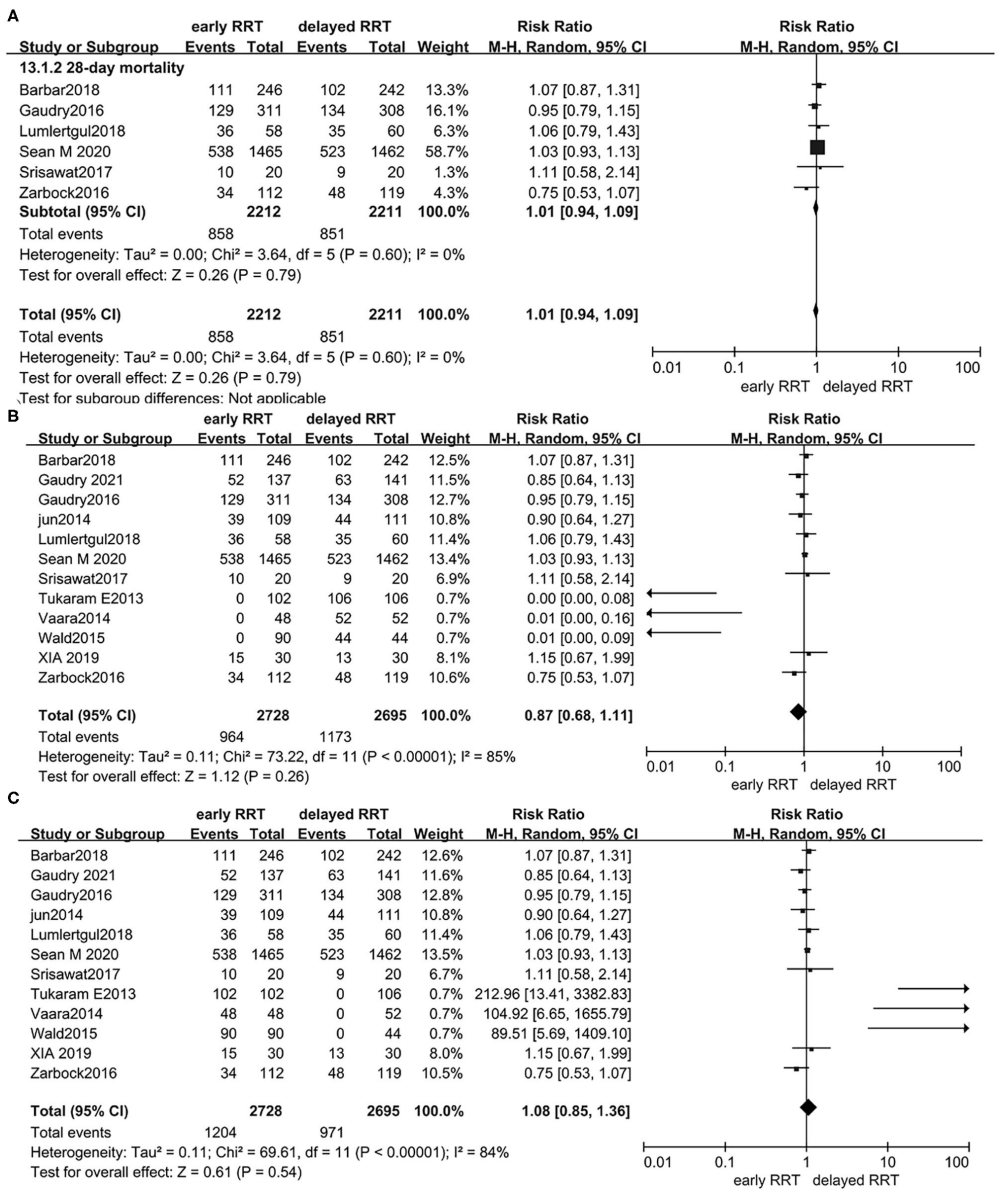
Forest plot for all-cause mortality at day 28 of sensitivity analysis by removing individual trials at a time with non-low bias in each domain **(A)**; best-worst-case scenario random-effects meta-analysis for the rate of all-cause mortality at day 28 **(B)**; and worst-best-case scenario random-effects meta-analysis for the rate of all-cause mortality at day 28 **(C)**.

To evaluate the potential effect of non-low bias studies on the robustness of data analysis, we performed a sensitivity analysis for the primary outcome by removing individual trials at a time with non-low bias in each domain. This sensitivity analysis produced similar results for the primary outcome (Figure 5A). Furthermore, we also conducted a sensitivity analysis by using the best-worst-case scenario analysis and worst-best-case scenario analysis to explore the influence of incomplete primary outcome data, which suggested that incomplete outcome data did not influence the results, either confirmed by the best-worst-case scenario analysis (RR 0.87, 95% CI: 0.68–1.11, *p* = 0.26, *I*^2^ = 85%) (Figure 5B) or by worst-best-case scenario analysis (RR 1.08, 95% CI: 0.85–1.36, *p* = 0.54, *I*^2^ = 84%) (Figure 5C), respectively.

### Secondary Outcomes

#### All-Cause Mortality (at Days 60 and 90, in ICU and in Hospital)

Definitions for all-cause mortality included all-cause mortality at day 60, day 90, in an ICU, and in a hospital. The conventional meta-analysis showed that all mortality outcomes did not differ between eRRT and dRRT (Supplementary Digital Content—Supplementary Figures S1A–D), TSA for all-cause mortality at day 60 showed that the cumulative *Z*-curve touched neither a traditional boundary nor trial sequential monitoring boundaries, indicating that further studies with larger sample size are needed to confirm this result. TSA for all-cause mortality at day 90, in an ICU, and in a hospital were consistent with those reported in mortality at day 28 (Supplementary Digital Content—Supplementary Figures S2A–D).

#### The Rate of RRT

The rate of RRT was reported in 10 trials, including 5,069 participants and 4,064 events (6, 12, 13, 15, 16, 30, 33–36). The result demonstrated that the rate of RRT significantly increased in eRRT (RR, 1.50, 95% CI: 1.28–1.76, *p* < 0.00001, *I*^2^ = 96%) (Supplementary Digital Content—Supplementary Figure S3A). TSA showed that the cumulative *Z*-curve touched the traditional boundary, but not for the sequential monitoring boundary, suggesting that further studies with a larger sample size are needed to rule out possible false positives (Supplementary Digital Content—Supplementary Figure S3B).

#### Ventilator-Free Days, Vasoactive Agent-Free Days, and the Rate of RRT Dependence at Day 28

There was also no significant difference in ventilator-free and vasoactive agents-free days between the two groups at day 28 (Supplementary Digital Content—Supplementary Figures S4A,B). About 7 of 12 trials (6, 12, 13, 16, 30, 34, 36) reported the rate of RRT dependence at day 28. The pooled analysis showed that the risk of RRT dependence at day 28 was not significantly different between the two groups (RR, 0.80, 95% CI: 0.50–1.29, *p* = 0.36, *I*^2^ = 58%) (Supplementary Digital Content—Supplementary Figure S5A), and the cumulative *Z*-curve did not touch a traditional boundary or trial sequential monitoring boundaries, indicating that further studies with a larger sample size are needed to confirm the result (Supplementary Digital Content—Supplementary Figure S5B).

#### The LOS of Survivors in ICU and Hospital

The meta-analysis from five of the included articles (6, 12, 15, 30, 33) showed that the LOS of survivors in an ICU (MD: −1.55 days; 95% CI: −3.08 to −0.02, *p* = 0.05, *I*^2^ =29%) (Supplementary Digital Content—Supplementary Figure S6A) and the LOS of survivors in a hospital were significantly decreased in eRRT (MD: −2.74 days; 95% CI: −4.93 to −0.55, *p* = 0.01, *I*^2^=14%) (Supplementary Digital Content—Supplementary Figure S6B).

### Adverse Events

Sean et al. (15) and Wald et al. (33) reported the total adverse events during the first 14 days, but the analysis was only relied on Sean's study because of Wald's too small trial. Sean et al. (15) reported that the adverse events included an association with renal-replacement therapy (hypotension, arrhythmia, seizure, bleeding, allergic reaction, decreased phosphate, decreased potassium, and decreased ionized calcium) and an association with the use of a dialysis catheter (pneumothorax or hemothorax, bleeding, thrombus, arterial puncture). The total adverse events of Wald et al. (33) include arrhythmia, hypotension, hemorrhage, hypocalcemia, ischemic bowel, and sepsis. Adverse events were significantly increased in eRRT (RR, 1.41, 95%CI: 1.22–1.63, *p* < 0.00001. Heterogeneity not applied) (Supplementary Digital Content—Supplementary Figure S7A), and it was confirmed by TSA (Supplementary Digital Content—Supplementary Figure S7B). However, the risk of bleeding, arrhythmia, and hypotension had no difference between the two groups (Supplementary Digital Content—Supplementary Figures S7C–E).

## Discussion

Conflicting results about the effect of eRRT vs. dRRT on patients with AKI exited for a long time, resulting in the optimal timing of RRT, which has been undecidable (37–39). Given the lack of quality evidence, more recently, two high-quality RCTs were published in NEJM (15) and Lancet (16), which compared the effects of eRRT vs. dRRT on critically ill patients with AKI. So, we did an updated systematic review with TSA.

In our meta-analysis, we found that there was no evidence to support the beneficial effects of eRRT on all-cause mortality at days 28 and 90, as well as in a hospital and in an ICU. These results were further confirmed by a sensitivity analysis or the TSA. Our result was consistent with some previous analyses (40, 41). In the analysis by Fayad et al. (42), the proportion of patients who died on day 28 did not significantly differ between the two groups. And, the other meta-analyses (40, 43) had the same results as did in Fayad et al. (42). However, some other meta-analyses have drawn the opposite conclusion. Zou et al. (44) did a meta-analysis, which included 15 studies with 1,479 patients and found that eRRT decreased 28-day mortality, especially when it was started within 24 h after cardiac surgery in patients with AKI. Wang et al. (45) selected 51 studies (including 10 RCTs) with a total of 8,179 patients with AKI showed that patients receiving eRRT had a 25% reduction in all-cause mortality compared to those receiving dRRT. These inconsistent results could be explained by several factors as follows. First, the sample sizes enrolled were quite large in the meta-analysis by Zou et al. and Wang et al., but a significant heterogeneity of the included studies could not be ignored, which may affect the robustness of the results. Second, study by Zou et al. was conducted in patients with AKI who had undergone cardiac surgery only. As we all know that most patients undergoing cardiac surgery were associated with cardiac insufficiency, and RRT can reduce generalized congestion and decrease cardiac load resulting in proving cardiac dysfunction.

These inconsistencies added further uncertainty about the efficacy of eRRT in critically ill patients with AKI. In a traditional meta-analysis, the positive results were easy to be changed with more evidence being accumulated (17, 46). Therefore, we performed TSA, of which the results were able to verify our traditional meta-analysis results: all-cause mortality at days 28 and 90, as well as in a hospital and in an ICU, and most importantly, to say no more studies were needed. While TSA for all-cause mortality at day 60 indicated that further studies with a larger sample size are needed to confirm this result, this may be related to the small sample size. In this respect, our analytic results of all-cause mortality at day 28, day 90, in a hospital, and in an ICU should be considered as convincing.

During our analysis, low degrees of heterogeneity were found in all-cause mortality at day 28 (*I*^2^ = 0%, *p* = 0.69). As we all know, in addition to statistical heterogeneity, it also includes clinical heterogeneity and methodological heterogeneity. Therefore, we conducted subgroup analyses based on study design, patient population, the modality of RRT, and the presence of sepsis at randomization, and found no substantial difference between the groups regarding mortality at day 28. Sensitivity analyses also showed that missing data did not change the primary outcome. However, the definitions of eRRT and dRRT in each included study were different, which prevents us from performing relevant heterogeneity analysis. In addition, we could not evaluate all possible causes of heterogeneity, which may affect the accuracy of our results.

In our analysis, an indicator of safety—the risk of total adverse events increased in eRRT and TSA also confirmed the result, but there was no significant difference in the risk of bleeding, arrhythmia, hypotension, and hyperkalemia. The TSA result required further studies are needed to confirm these results. The inconsistency may be due to the inclusion of different studies for the analysis of these two results. Therefore, it is expected that the same studies are used simultaneously for these two indicators.

Our meta-analysis has the following strengths: (1) We prespecified our analytical plan and registered the study protocol with INPLASY to ensure the methodological quality of the review. (2) We selected RCTs published only in the last 10 years and included the latest published high-quality RCTs (15, 16). (3) Considering clinical heterogeneity especially due to differences in the definition of eRRT and dRRT, we performed a sensitivity analysis with the “best-worst-case” scenario and “worst-best-case” scenario to assess the possible impact of missing data on the robustness of results and performed subgroup analyses to explore eventual heterogeneity. (4) We also conducted TSA to ascertain the trend of uncertain outcomes and provide “futility boundaries” that can draw invalid conclusions as soon as possible, preventing researchers from spending a lot of resources on unneeded research.

There were several limitations in our analysis. First, the heterogeneity of studies, particularly the definition of eRRT and dRRT was quite various and might affect the accuracy of our results. Second, there is an inability to assess all possible causes of heterogeneity, the decision regarding the optimal timing of RRT lack of objective basis. Third, the total adverse events and other specific adverse events showed contradictory results, the inconsistency possibly due to the inclusion of different studies for analysis.

## Conclusion

Early RRT strategy provided no significant survival benefit for ill patients with AKI compared with the dRRT strategy, and TSA indicated that no more studies were needed to confirm it.

## Data Availability Statement

All data generated or analyzed during this study are included in this published article (and its Supplementary Material), further inquiries can be directed to the corresponding author

## Author Contributions

CX contributed to the study concept and design, drafting of the manuscript. CX, JX, and YC contributed to the acquisition, analysis, and interpretation of data, and statistical analysis. QL, WL, TH, SL, and DG helped in data arrangement. FS contributed to study concept, supervision, and organizing the final manuscript. All authors contributed to the article and approved the submitted version.

## Conflict of Interest

The authors declare that the research was conducted in the absence of any commercial or financial relationships that could be construed as a potential conflict of interest.

## Publisher's Note

All claims expressed in this article are solely those of the authors and do not necessarily represent those of their affiliated organizations, or those of the publisher, the editors and the reviewers. Any product that may be evaluated in this article, or claim that may be made by its manufacturer, is not guaranteed or endorsed by the publisher.

## Supplementary Material

Supplementary Figure S1 Forest plot for all-cause mortality at day 60. The size of each square represents the proportion of information provided by each study **(A)**; forest plot for all-cause mortality at day 90 **(B)**; forest plot for all-cause mortality in an intensive care unit (ICU) **(C)**; and forest plot for all-cause mortality in a hospital **(D)**.

Supplementary Figure S2 Trial sequential analysis (TSA) for all-cause mortality at day 60 **(A)**; TSA for all-cause mortality at day 90 **(B)**; TSA for all-cause mortality in an ICU **(C)**; and TSA for all-cause mortality in a hospital **(D)**.

Supplementary Figure S3 Forest plot for the rate of renal replacement therapy (RRT) during the study period **(A)** and TSA for the rate of RRT during the study period **(B)**.

Supplementary Figure S4 Forest plot for ventilator-free days at day 28 **(A)** and forest plot for vasoactive agent-free days at day 28 **(B)**.

Supplementary Figure S5 Forest plot for the rate of RRT dependence at day 28 **(A)** and TSA for the rate of RRT dependence at day 28 **(B)**.

Supplementary Figure S6 Forest plot for the length of stay (LOS) of survivors in ICU **(A)** and forest plot for the LOS of the survivors in a hospital **(B)**.

Supplementary Figure S7 Forest plot for the rate of total adverse events **(A)**; TSA for the rate of total adverse events **(B)**; forest plot for the rate of bleeding **(C)**; forest plot for the rate of arrhythmia **(D)**; and forest plot for the rate of hypotension **(E)**.

## Abbreviations

eRRT: early renal replacement therapy
AKI: acute kidney injury
TSA: trial sequential analysis
RCTs: randomized controlled trials
dRRT: delayed renal replacement therapy
RR: risk ratio
CI: confidence interval
GRADE: Grading of Recommendations, Assessment, Development and Evaluations
ICU: intensive care unit
RRT: renal replacement therapy
LOS: the length of stay
RRR: relative risk reduction
SOFA: Sequential Organ Failure Assessment
APACHE II: Acute Physiology and Chronic Health Evaluation
FST: furosemide stress test
pNGAL: plasma neutrophil gelatinase associated lipocalin.
