# Supplementary figures and images for: The Efficacy and Safety of Early Renal Replacement Therapy in Critically Ill Patients With Acute Kidney Injury: A Meta-Analysis With Trial Sequential Analysis of Randomized Controlled Trials
Source: Front Med (Lausanne). 2022 Feb 21;9:820624. doi: 10.3389/fmed.2022.820624 (PMC8898954; doi:10.3389/fmed.2022.820624)

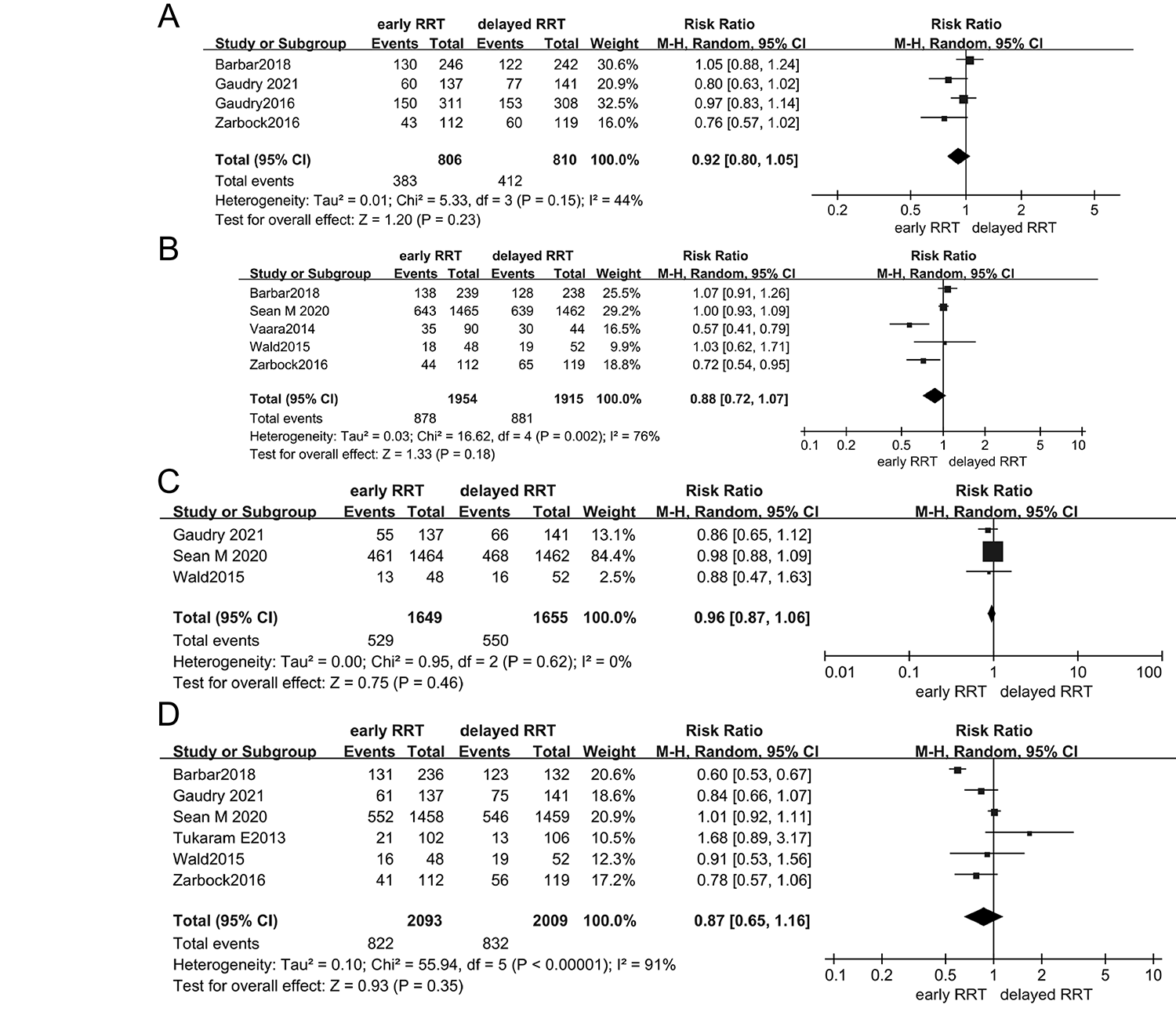

Supplement: Supplementary Figure S1 — Forest plot for all-cause mortality at day 60. The size of each square represents the proportion of information provided by each study (A); forest plot for all-cause mortality at day 90 (B); forest plot for all-cause mortality in an intensive care unit (ICU) (C); and forest plot for all-cause mortality in a hospital (D). [file Data_Sheet_1.ZIP › Fig. S1.tif]

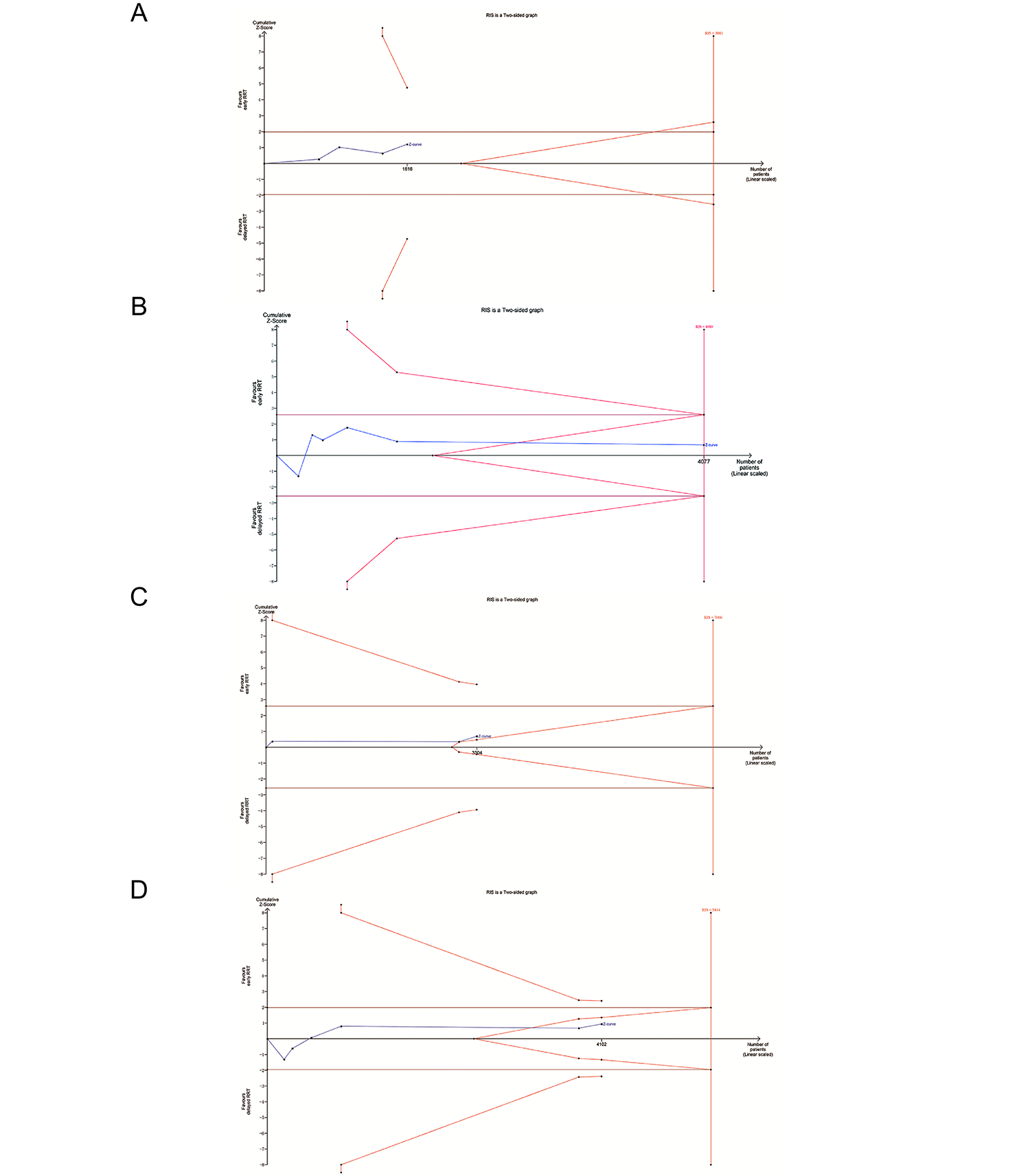

Supplement: Supplementary Figure S1 — Forest plot for all-cause mortality at day 60. The size of each square represents the proportion of information provided by each study (A); forest plot for all-cause mortality at day 90 (B); forest plot for all-cause mortality in an intensive care unit (ICU) (C); and forest plot for all-cause mortality in a hospital (D). [file Data_Sheet_1.ZIP › Fig. S2.tif]

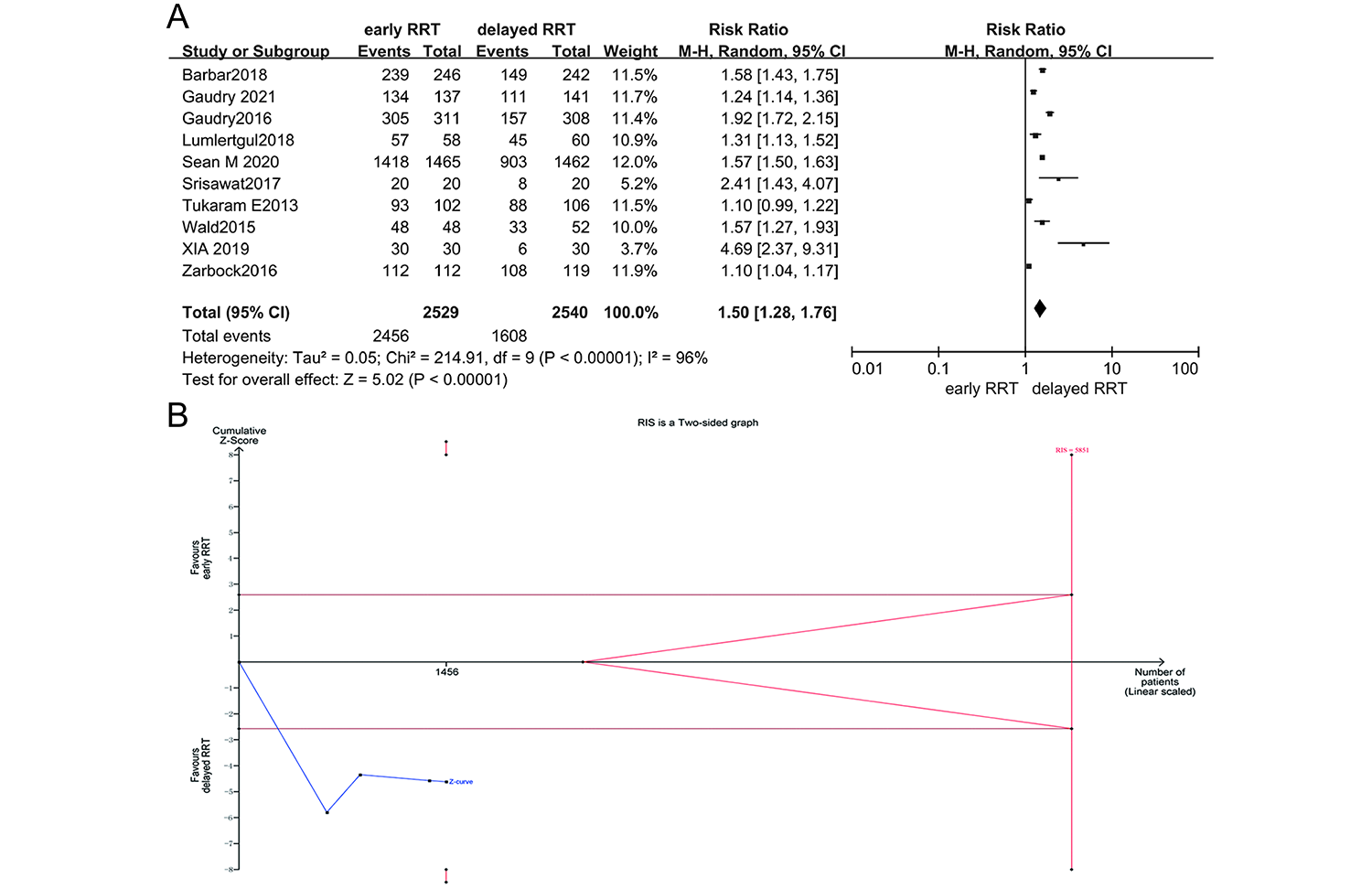

Supplement: Supplementary Figure S1 — Forest plot for all-cause mortality at day 60. The size of each square represents the proportion of information provided by each study (A); forest plot for all-cause mortality at day 90 (B); forest plot for all-cause mortality in an intensive care unit (ICU) (C); and forest plot for all-cause mortality in a hospital (D). [file Data_Sheet_1.ZIP › Fig. S3.tif]

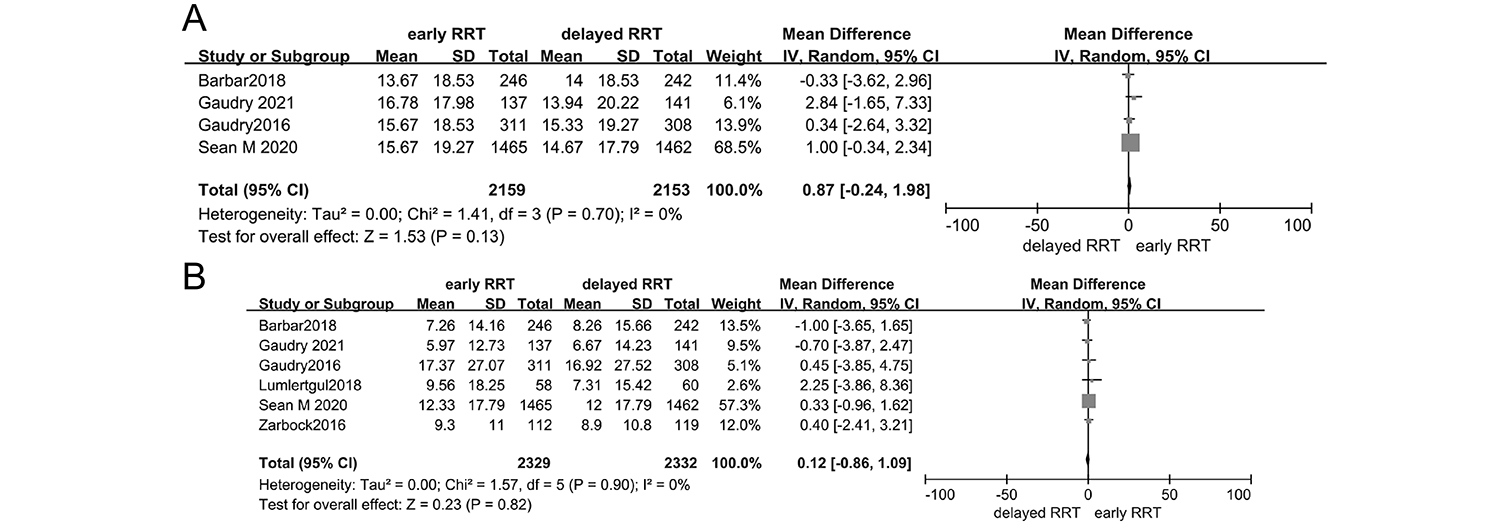

Supplement: Supplementary Figure S1 — Forest plot for all-cause mortality at day 60. The size of each square represents the proportion of information provided by each study (A); forest plot for all-cause mortality at day 90 (B); forest plot for all-cause mortality in an intensive care unit (ICU) (C); and forest plot for all-cause mortality in a hospital (D). [file Data_Sheet_1.ZIP › Fig. S4.tif]

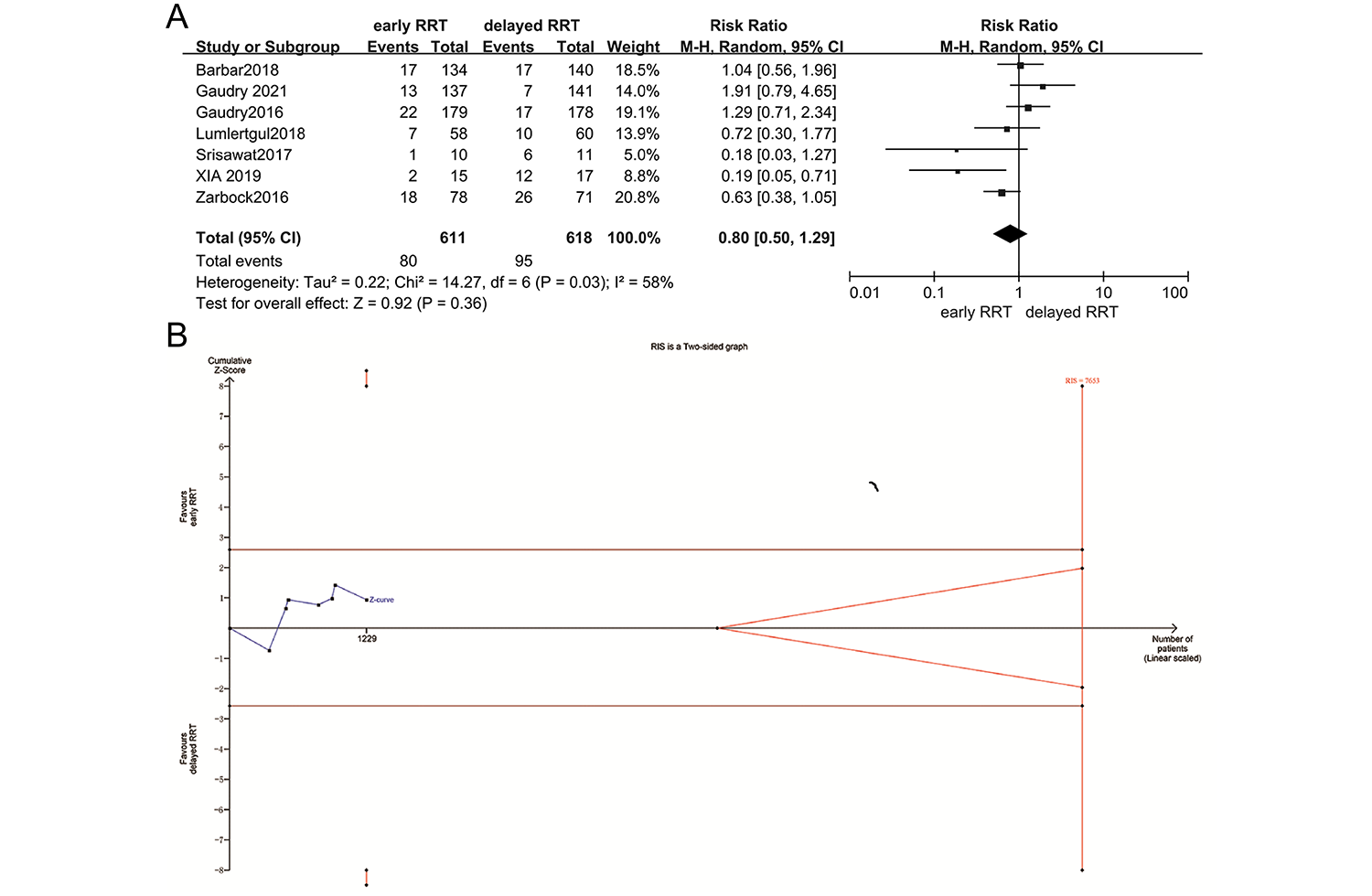

Supplement: Supplementary Figure S1 — Forest plot for all-cause mortality at day 60. The size of each square represents the proportion of information provided by each study (A); forest plot for all-cause mortality at day 90 (B); forest plot for all-cause mortality in an intensive care unit (ICU) (C); and forest plot for all-cause mortality in a hospital (D). [file Data_Sheet_1.ZIP › Fig. S5.tif]

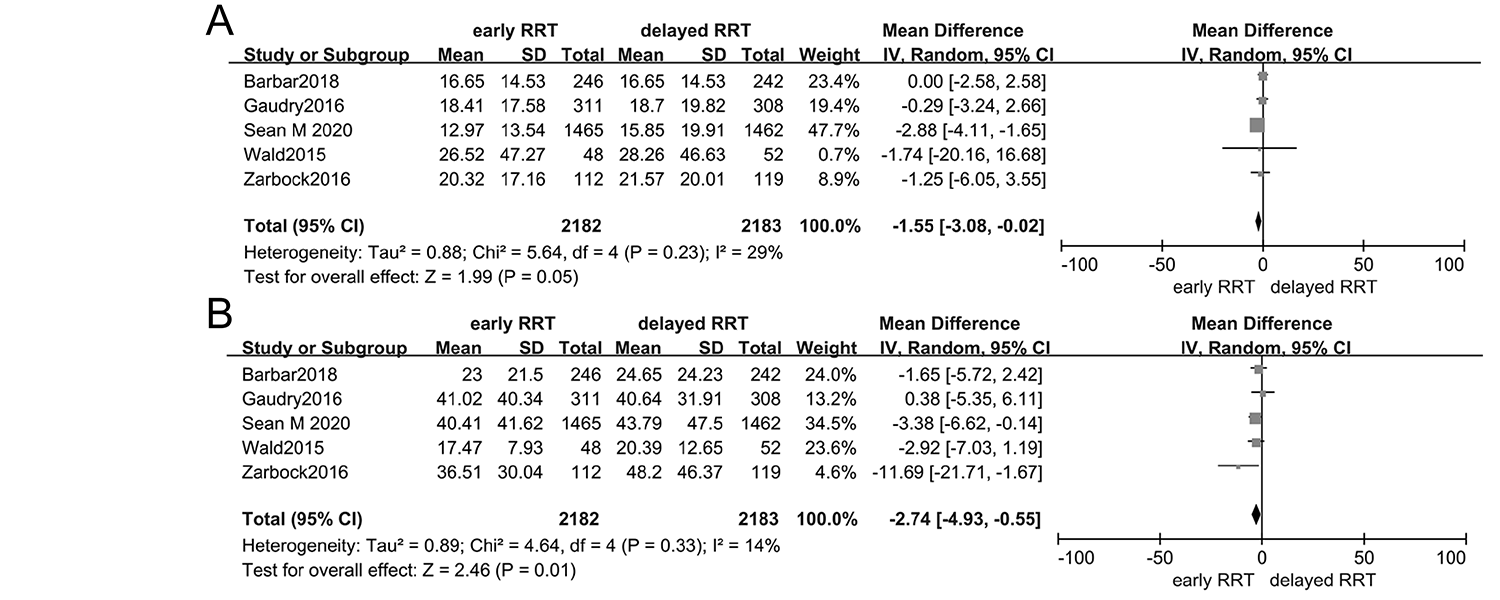

Supplement: Supplementary Figure S1 — Forest plot for all-cause mortality at day 60. The size of each square represents the proportion of information provided by each study (A); forest plot for all-cause mortality at day 90 (B); forest plot for all-cause mortality in an intensive care unit (ICU) (C); and forest plot for all-cause mortality in a hospital (D). [file Data_Sheet_1.ZIP › Fig. S6.tif]

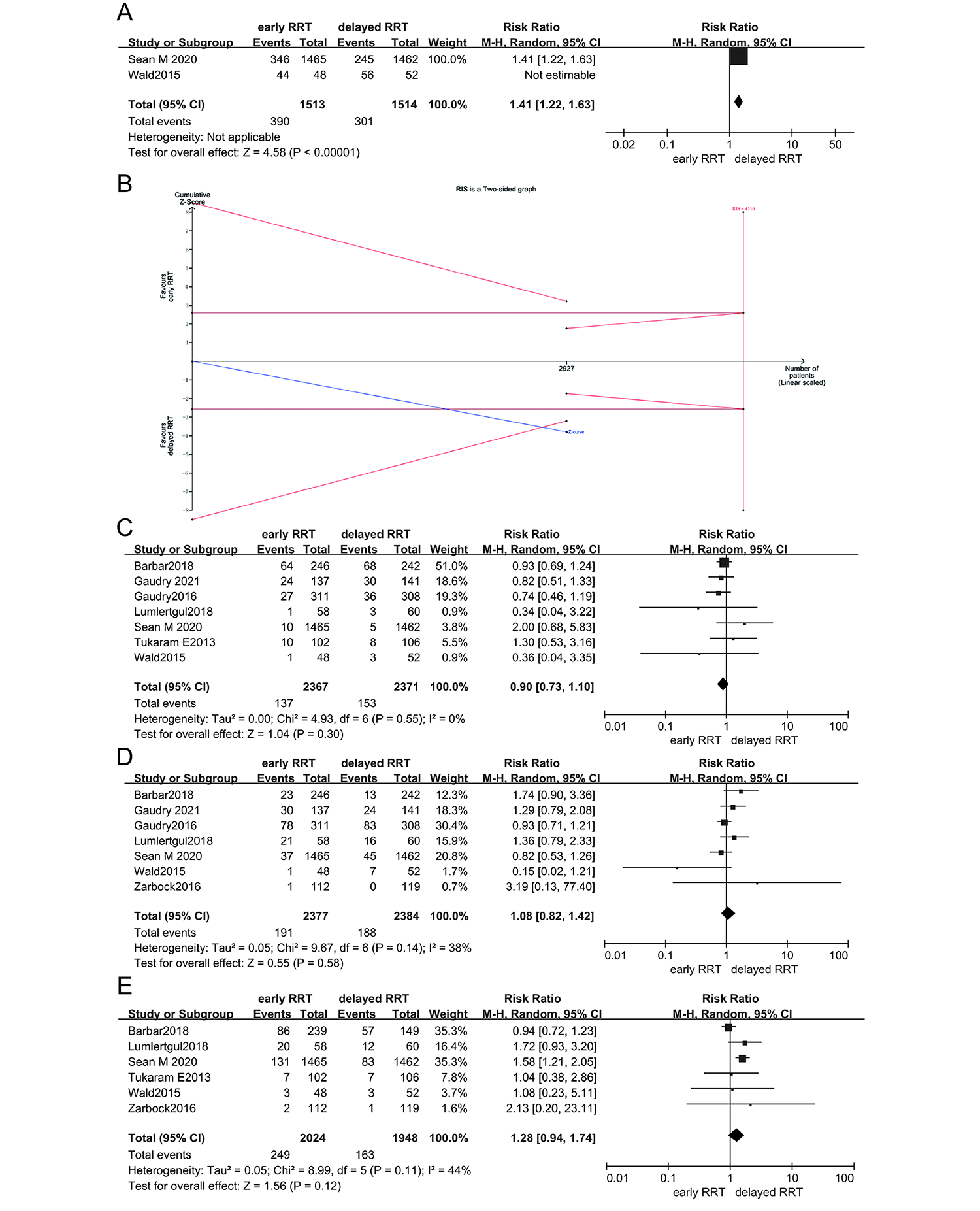

Supplement: Supplementary Figure S1 — Forest plot for all-cause mortality at day 60. The size of each square represents the proportion of information provided by each study (A); forest plot for all-cause mortality at day 90 (B); forest plot for all-cause mortality in an intensive care unit (ICU) (C); and forest plot for all-cause mortality in a hospital (D). [file Data_Sheet_1.ZIP › Fig. S7.tif]
