# Supplementary material for: The Efficacy and Safety of Early Renal Replacement Therapy in Critically Ill Patients With Acute Kidney Injury: A Meta-Analysis With Trial Sequential Analysis of Randomized Controlled Trials
Source: Front Med (Lausanne). 2022 Feb 21;9:820624. doi: 10.3389/fmed.2022.820624 (PMC8898954; doi:10.3389/fmed.2022.820624)
Supplement: Supplementary file 3 [file Table_2.DOCX]

EMBase

1. acute kidney failure/

2. (acute kidney failure or acute renal failure).tw.

3. (acute kidney injury or acute renal injury).tw.

4. (acute kidney insufficiency or acute renal insufficie$).tw.

5. acute tubular necrosis.tw.

6. (ARI or AKI or ARF or AKF or ATN).tw.

7. or/1-6

8. continuous renal replacement therapy/ or exp renal replacement therapy/

9. (continuous adj3 hemofiltration).tw.

10. (continuous adj3 hemodiafiltration).tw.

11. (continuous adj3 h?emodialysis).tw.

12. continuous ultrafiltration.tw.

13. (CVVH or CVVHDF or CVVHD or SCUF or CRRT).tw.

14. (intermittent h?emodialysis or IHD).tw.

15. renal replacement therap$.tw.

16. (sustained low efficiency dialysis or SLED).tw.

17. (extended daily dialysis or EDD).tw.

18. hemoperfusion.tw.

19. or/8-18

CENTRAL

1.MeSH descriptor: [Acute Kidney Injury] explode all trees

2."acute kidney failure":ti,ab,kw OR "acute renal failure":ti,ab,kw in Trials

3."acute kidney injury":ti,ab,kw OR "acute renal injury":ti,ab,kw in Trials

4."acute kidney insufficiency":ti,ab,kw OR "acute renal insufficiency":ti,ab,kw in Trials

5."acute tubular necrosis":ti in Trials

6.(ARI or AKI or ARF or AKF or ATN):ti,ab,kw in Trials

7.#1 or #2 or #3 or #4 or #5 or #6 in Trials

8.MeSH descriptor: [Renal Replacement Therapy] explode all trees

9.continuous near/2 haemofiltration:ti,ab,kw in Trials

10.continuous near/2 haemodiafiltration:ti,ab,kw in Trials

11.continuous near/2 haemodialysis:ti,ab,kw in Trials

12.continuous next ultrafiltration:ti,ab,kw in Trials

13.CVVH or CVVHDF or CVVHD or SCUF or CRRT:ti,ab,kw in Trials

14.renal replacement therap*:ti,ab,kw in Trials

15.intermittent hemodialysis or intermittent haemodialysis:ti,ab,kw (Word variations have been searched)

16."sustained low efficiency dialysis" or SLED:ti,ab,kw (Word variations have been searched)

17."extended daily dialysis" or EDD:ti,ab,kw (Word variations have been searched)

18.hemoperfusion:ti,ab,kw (Word variations have been searched)

19.{or #8‐#18}

20{and #7, #19}

Pubmed

1.MeSH descriptor: [Acute Kidney Injury] explode all trees

2."acute kidney failure":ti,ab,kw OR "acute renal failure":ti,ab,kw in Trials

3."acute kidney injury":ti,ab,kw OR "acute renal injury":ti,ab,kw in Trials

4."acute kidney insufficiency":ti,ab,kw OR "acute renal insufficiency":ti,ab,kw in Trials

5."acute tubular necrosis":ti in Trials

6.(ARI or AKI or ARF or AKF or ATN):ti,ab,kw in Trials

7.#1 or #2 or #3 or #4 or #5 or #6 in Trials

8.MeSH descriptor: [Renal Replacement Therapy] explode all trees

9.continuous near/2 haemofiltration:ti,ab,kw in Trials

10.continuous near/2 haemodiafiltration:ti,ab,kw in Trials

11.continuous near/2 haemodialysis:ti,ab,kw in Trials

12.continuous next ultrafiltration:ti,ab,kw in Trials

13.CVVH or CVVHDF or CVVHD or SCUF or CRRT:ti,ab,kw in Trials

14.renal replacement therap*:ti,ab,kw in Trials

15.intermittent hemodialysis or intermittent haemodialysis:ti,ab,kw (Word variations have been searched)

16."sustained low efficiency dialysis" or SLED:ti,ab,kw (Word variations have been searched)

17."extended daily dialysis" or EDD:ti,ab,kw (Word variations have been searched)

18.hemoperfusion:ti,ab,kw (Word variations have been searched)

19.{or #8‐#18}

20{and #7, #19}

RCT

("Randomized Controlled Trial" [Publication Type] OR "Controlled Clinical Trial" [Publication Type] OR "Clinical Trials as Topic"[Mesh: NoExp] OR randomized[Title/Abstract] OR placebo [Title/Abstract] OR randomly[Title/Abstract] OR trial[Title/Abstract]) NOT ("Animals"[Mesh] NOT "Humans"[Mesh])

ClinicalTrials.gov

Condition or disease :Acute Kidney Injury OR acute kidney failure OR acute renal failure OR acute kidney insufficiency OR acute renal insufficiency OR ARI OR AKI OR ARF OR AKF

LILACS

1.acute kidney failure/

2. acute kidney failure or acute renal failure) tw

3. acute tubular necrosis.tw.

4. or/1-3

5. continuous renal replacement therapy/

6. (continuous venovenous haemofiltration or continuous venovenous haemofiltration) tw.

7. (continuous venovenous haemodiafiltration or continuous venovenous haemodiafiltration) tw.

8. (continuous venovenous haemodialysis or continuous venovenous haemodialysis) tw.

9. or/5-8

10. 4 AND 9

Grey literature :

OpenGrey ([www.opengrey.eu](http://www.opengrey.eu))

Acute Kidney Injury OR acute kidney failure OR acute renal failure OR acute kidney insufficiency OR acute renal insufficiency OR ARI OR AKI OR ARF OR AKF

BASE (Bielefeld Academic Search Engine)

Acute Kidney Injury OR acute kidney failure OR acute renal failure OR acute kidney insufficiency OR acute renal insufficiency OR ARI OR AKI OR ARF OR AKF
